# Phasic Alertness and Multisensory Integration Contribute to Visual Awareness of Weak Visual Targets in Audio-Visual Stimulation under Continuous Flash Suppression

**DOI:** 10.3390/vision6020031

**Published:** 2022-06-03

**Authors:** Anna Matilda Helena Cederblad, Juho Äijälä, Søren Krogh Andersen, Mary Joan MacLeod, Arash Sahraie

**Affiliations:** 1 Division of Biology and Biological Engineering, California Institute of Technology, Pasadena, CA 91125, USA; 2 School of Psychology, University of Aberdeen, Aberdeen AB24 3FX, UK; juho.aijala.19@alumni.ucl.ac.uk (J.Ä.); m.j.macleod@abdn.ac.uk (M.J.M.); a.sahraie@abdn.ac.uk (A.S.); 3 Department of Psychology, University of Southern Denmark, 5230 Odense, Denmark; skandersen@health.sdu.dk; 4 The Institute of Medical Sciences, University of Aberdeen, Aberdeen AB24 3FX, UK

**Keywords:** multisensory stimulation, multisensory integrations, alertness, phasic alertness, visual awareness

## Abstract

Multisensory stimulation is associated with behavioural benefits, including faster processing speed, higher detection accuracy, and increased subjective awareness. These effects are most likely explained by multisensory integration, alertness, or a combination of the two. To examine changes in subjective awareness under multisensory stimulation, we conducted three experiments in which we used Continuous Flash Suppression to mask subthreshold visual targets for healthy observers. Using the Perceptual Awareness Scale, participants reported their level of awareness of the visual target on a trial-by-trial basis. The first experiment had an audio-visual Redundant Signal Effect paradigm, in which we found faster reaction times in the audio-visual condition compared to responses to auditory or visual signals alone. In two following experiments, we separated the auditory and visual signals, first spatially (experiment 2) and then temporally (experiment 3), to test whether the behavioural benefits in our multisensory stimulation paradigm could best be explained by multisensory integration or increased phasic alerting. Based on the findings, we conclude that the largest contributing factor to increased awareness of visual stimuli accompanied by auditory tones is a rise in phasic alertness and a reduction in temporal uncertainty with a small but significant contribution of multisensory integration.

## 1. Introduction

When sensory stimulation–such as visual and auditory signals–is presented at approximately the same time and from the same location, the signals are often bound together [1]. This multisensory stimulation is associated with specific behavioural benefits [1,2,3,4] such as higher accuracy and identification of targets, both in detection [2,3] and reporting the target location [5,6] and in identifying the second target in an attentional blink paradigm [7,8]. An additional benefit from using multisensory stimulation regards the subjectively reported awareness of specifically visual stimuli —to clarify, it has been found that stimulation in one sensory modality can modulate the perceived intensity or subjective awareness of a signal presented to another modality [3,4,5]; this is also true for patients suffering from hemianopia or neglect [9] (but sadly does not improve detection performance in patients suffering from a combination of both hemianopia and neglect). For example, in an experiment in which healthy participants were instructed to rate the intensity of a red LED light, it was found that when the light was presented with an auditory stimulus, the participants reported that they perceived the LED light to be more intense compared to when it was presented alone [4]. Another example of a context in which multisensory stimulation has proven to be beneficial is in processing speed, which is evident in faster reaction times in detection paradigms [10] such as in the Redundant Signal Effect (RSE) paradigm [11]. RSE entails that reaction times for target detection are faster when multiple signals coincide compared to when each signal is presented in isolation [11]. RSE occurs for signals that are within one sensory modality, such as with only visual targets [12], or with presentation across different modalities [10,13]. For multisensory stimulation, this means that responses related to the detection of a sensory signal is quicker when the signal is presented in combination with a signal of another sensory modality [10,13].

The evidence for behavioural benefits of multisensory stimulation is consistent, nevertheless, the mechanism behind the effect is not yet clearly understood. There are two mechanisms that have been suggested as the likely cause behind the beneficial effects of multisensory stimulation. These are multisensory integration, or a heightened state of alertness or arousal. Multisensory integration refers to sensory signals being processed together on a neuronal level [1,6,14,15]. There are three established principles of multisensory integration [1,16]. The first principle regards the spatial relationship between the signals in that the more spatially congruent signals are, the stronger the effect is (i.e., coming from the same location). The second principle regards the temporal relationship where the signals should ideally be simultaneous to generate the strongest effect. The third is the principle of Inverse Effectiveness, which means that the difference seen by combining signals is stronger if one of the signals is weak, and the effect increases with decreasing signal strength of the weaker target [4].

The Continuous Flash Suppression (CFS) paradigm can be utilised to reduce subjective awareness of visual targets [17]. In CFS, the dominant eye of an observer is shown a flashing Mondrian through a binocular rivalry display,, and the other eye sees the stimulus of interest. Because the CFS-mask is a powerful and dynamic stimulation, it suppresses information shown to the second eye [17]. For example, Aller et al. [5] conducted an experiment with audio-visual targets under CFS where the participant was tasked with locating the visual target. They concluded that multisensory integration was the determining mechanism behind whether the weak targets were brought into awareness by the help of a spatially congruent tone. Furthermore, their participants reported a higher instance of awareness of visual targets for congruent than for incongruent audio-visual targets.

Further support of the multisensory integration theory comes from the discovery of bimodal neurons that respond to two different sensory modalities, and trimodal neurons that respond to three modalities [1]. Additionally, a subsection of unimodal neurons–coined subthreshold multisensory neurons–respond solely to signals in one modality, but their response is modulated by the presence and intensity of signals in another modality [14,15]. For example, some neurons in the extrastriate visual cortex of cats have been found to increase their firing rates when visual stimuli are accompanied by auditory stimulation [14]. These visual neurons did however not respond to auditory stimulation on its own, but their firing rates increased with increased intensity of the auditory stimulation [15].

The second explanation for behavioural benefits from multimodal stimulation is that the effect comes from an increase in alertness [18,19,20]. Li et al. [19] argued that phasic alertness, referring to a rise in alertness from a non-specific activation, can explain behavioural benefits from multisensory stimulation. They found that warning cues, which could be auditory or visual, were associated with improved performance in temporal precision judgements of whether visual and auditory targets occurred simultaneously or whether one preceded another, and that this effect was likely to be largely independent of temporal expectancy. Furthermore, introducing an alerting auditory signal to a visual task can improve performance both in terms of reaction time and accuracy in an orientation discrimination task [20]. Using a visual orientation discrimination task, Kusnir et al. [20] showed that an alerting tone preceding a near-threshold visual target was associated with higher discrimination accuracy only if there was temporal uncertainty of the visual and auditory target onset. This was compared to a condition where the target onset was predictable, where there was no effect on accuracy with the presence of a tone. They argued that the improved performance seen in the condition with the tone were due to phasic alerting improving the conscious perception of visual targets of individualised near-threshold detection. Another line of evidence regarding specific properties of phasic alertness and its effect on performance on a perceptual task comes from Weinbach and Henik [21], who found that adding a tone as an alerting signal increased attention to salient visual information, sometimes to the detriment of task performance. They used hierarchical stimulus [22] in the form of a large arrow consisting of smaller arrows, and instructed participants to engage in global or local processing. The large and small arrows could be pointing in the same or opposite directions. If all the small arrows were the same colour (making the big arrow consistent), this was considered the Global Salient presentation. If the smaller arrows building the big arrow were of two different colours, this was considered a local salient presentation. They found that by adding an auditory stimulus to the presentation, the participants’ attention was directed to the most salient information in the display. Thus, if participants were instructed to engage in global processing but the most visually salient event was local, there was a larger local interference with an alerting tone and vice versa. When the salient information was adhered to the current task, the alerting tone was associated with better performance. To conclude, there is evidence for both multisensory integration and increased arousal or phasic alerting as possible mechanisms behind the behavioural benefits seen in multisensory stimulation. However, increased phasic alerting is only associated with improved task performance if attention is not grabbed by highly salient non-relevant information.

Here, we report three experiments where we examined the behavioural effects of multisensory stimulation and reported subjective awareness. In the first experiment, we investigated the effects on reaction time and self-reported awareness in audio-visual interactions. The hypothesis regarding behavioural benefits from multisensory stimulation was two-fold. First, that there would be faster responses for the audio-visual compared to the audio only presentation, and secondly that there would be a higher instance of subjective awareness of the visual target for the audio-visual presentation compared to when the visual target was presented alone. Findings from experiment 1 support both hypotheses, and we could thus confirm that we observed the expected benefits of multisensory presentations. However, the explanation behind the mechanism of these behavioural improvements were not clear. Aller et al. [5] conducted an experiment with a similar paradigm where weak visual targets were presented underneath a CFS-mask in three different locations (centre, to the left, or to the right of the centre) with a tone that could be spatially congruent or incongruent with the visual target. They found the best performance and higher awareness for spatially congruent presentations and concluded that their findings were most likely best explained by multisensory integration. However, they only used two lateralised locations from where the tone and the visual target could appear, and it is likely that this was an overly simplified spatial cueing (target on the left or right of the fixation). They found that spatial congruency was associated with higher probability that the visual targets were perceived. When participants reported to not have seen the visual target, the weight of the sound location was the largest, perhaps due to the possibility that in the absence of any other evidence, the participants reported the location of the auditory target. The findings by Aller et al. [5] are intriguing but leave some unanswered questions as to the detailed nature of multisensory stimulation and its effect on visual awareness. We hypothesised that the behavioural benefits from audio-visual presentations were due to multisensory integration, and based on the spatial principle of multisensory integration [1], that better performance would be associated with presentations where a visual target and an auditory tone came from the same location compared to mirror-opposite presentations. We therefore utilised the spatial principle of multisensory integration in the second experiment where we examined the audio-visual spatial relationships and detection accuracy of the visual targets. The findings of experiment 2 were inconclusive in the context of determining whether multisensory integration was the underlying mechanism for audio-visual behavioural benefits because the performance was similar when the tone was spatially relevant and spatially irrelevant (coming from the centre of the display, but not misleading as in coming from another quadrant of the display). At this stage, we considered that the behavioural benefits could be explained by multisensory integration or an increase in phasic alerting. In the third experiment we therefore separated the targets temporally, and using the temporal principle of multisensory integration [1] we hypothesised that if the performance in the visual detection task was better for displays where the tone preceded or was simultaneous with the visual target, the most likely explanation would be an increase in phasic alertness. If the performance was the best for the simultaneous presentation, then the best support would be for multisensory integration. The findings point to a rise in alertness as a major contributing factor to reported awareness with a small but significant contribution of multisensory integration.

## 2. General Methods

In all three experiments, the participants were greeted by one or two experimenters. They were instructed about the experimental task and consented to participate. Ethical approval was granted for all experiments by the School of Psychology Ethics Committee. Participants were recruited through word-of-mouth at the University of Aberdeen, and some were recruited through an online study-for-course credits source for undergraduate psychology students at the University of Aberdeen.

**Figure 1:**
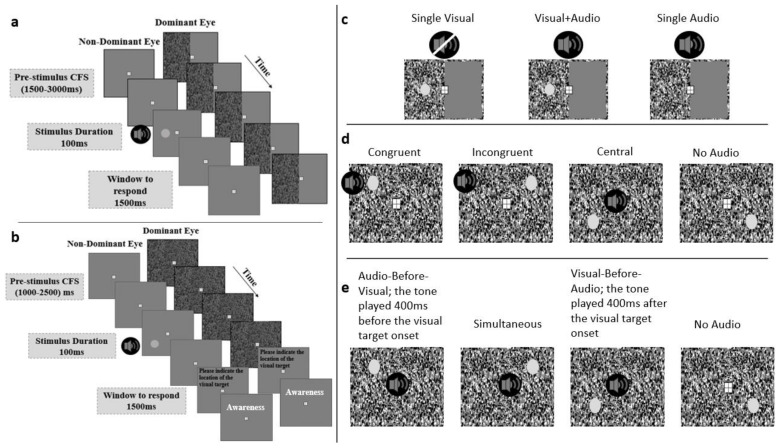
Schematic illustrations of the trial structure of experiment 1 (**a**), and experiments 2 and 3 (**b**). In (**c**–**e**), all conditions are listed from left to right. Experiment 1 had three conditions: Single Visual, Visual + Audio, and Single Audio (**c**). Experiment 2 had four conditions where the auditory tone and visual target locations were either: Congruent, Incongruent, non-informative audio (Central), or No Audio. Experiment 3 had four conditions: the auditory tone either preceded by 400 ms (Audio-Before-Visual), occurred simultaneously, or succeeded the visual target by 400 ms (Visual-Before-Audio), or the tone was absent (No Audio).

During the experiments, participants were placed in a head-and-chin rest in front of a PC monitor. The monitor was a generic LCD PnP monitor (AOC), which had a resolution of 1920 × 1080 pixels, and a refresh rate of 100Hz. The PC was an HP EliteDesk 800 G4 SFF. Participants viewed the monitor through a mirror-stereoscope to display different images to each eye. The “hole in the hand test” [23] was applied to determine participants’ eye dominance. CFS [17] was used to render part of the display outside participants’ conscious awareness. During the experiment, the dominant eye was presented with a dynamic flashing Mondrian mask (10 Hz), which was applied to half of the visual field in experiment 1, and to the entire visual field in Experiments 2 and 3 (Figure 1a,b). The background and the visual target were displayed to the non-dominant eye. In Experiment 1, participants pressed the spacebar on a computer keyboard in front of them when they detected targets (visual and auditory). In Experiments 2 and 3, participants were asked to use four keys placed in a trapezoid (keys “F”, “V”, “J”, “N”) on a computer keyboard to report the location of the visual target that was presented in one of the four quadrants of the display. The keys corresponded to the location where the visual target could appear on the screen: top left, bottom left, top right, bottom right. The presentation of the visual targets of Experiments 2 and 3 were randomly assigned across the four locations.

The stimuli of the three experiments were auditory and visual. See Figure 1 for a schematic display of the stimulus arrangements and the conditions of the three experiments as well as the timing within trials. The visual targets were 1.16^o^ in diameter and the viewing distance was 39.5 cm. The fixation cross was 1.31^o^ wide, presented at the centre on both left and right eye images, and the targets were placed 1.7^o^ from the centre of the display. The auditory target was a pure tone of 1 kHz. The visual and auditory targets were presented for the same duration (100 ms). Playback of the tones was initiated through an AudioFile, a short-latency trigger box (Cambridge Research Systems Limited) [24], and played through speakers spatially coinciding with the masked visual target in Experiment 1. In Experiment 2, there were five possible locations from which the tone could come from. It was either in one of the four quadrants or in the centre of the display (all four speakers co-located with the quadrants of the display playing at the same time). In the third experiment, the tone always came from the centre of the display but with different temporal onsets relative to the visual target.

The experiments were run in MATLAB version R2017a, the visual stimuli were generated in GIMP (version 2.8.22 GNU Image Manipulation Program). The background display of the monitor was grey and had a luminance of 40 cd/m^2^. All luminance measurements for targets and backgrounds were carried out using a Luminance meter (LS-100, Minolta, Japan).

### 2.1. Threshold Measurements

First, the participants’ contrast detection threshold for a visual target under CFS was measured. The visual stimuli used were the same in size, shape, and location as the masked visual targets described for the main experiments. In each trial of the threshold measurement, a visual target appeared under the CFS-mask and increased in contrast from the background in steps of 1% up to the maximum of 30% above the background grey (40 cd/m^2^) background. Participants were instructed to press the spacebar as soon as they detected the target. In Experiment 1, participants’ detection threshold was measured for one location in the mask (the same as the masked target would appear in). In Experiments 2 and 3, thresholds were measured for all four locations that targets could be presented (one visual target in each quadrant). All participants completed one practice block of the threshold measurement task. Visual targets were switched on and off (100 ms On –200 ms Off) repeatedly with the target contrast increasing by 1% at each cycle, until the participant pressed the spacebar or a maximum of 30% contrast was achieved. The contrast at which the visual target was detected (broke through suppression) was the average contrast at detection for 20 trials at each location and for each participant. These contrasts were collapsed across all visual target locations, and the result was considered the detection threshold for that participant. To ensure that the stimuli in the main experiment were set to an individualised subthreshold value, the contrast of the visual targets was set to 3db below the participants’ individualised thresholds. The mean detection thresholds for target contrast across participants were 8.61% (*SD* = 1.9%), 7.91% (*SD* = 1.31%), and 8.76% (*SD* = 2.44%) for experiments 1–3, respectively. The mean contrast used for the targets for each experiment were 4.7% (*SD* = 0.93%), 4.24% (*SD* = 0.6%), and 4.6% (*SD* = 1.22%) for Experiment 1–3, respectively.

### 2.2. Subjective Awareness Rating 

Participants verbally reported their awareness of masked visual targets on a trial-by-trial basis. Their responses were entered onto a separate computer keyboard by the experimenter during testing. The scale used for reporting subjective awareness was the Perceptual Awareness Scale (PAS), which is a 4-point scale that ranges from 1—“no experience”, 2—“brief glimpse”, 3—“almost clear experience”, 4—“clear experience” [25].

### 2.3. Defining Invalid Trials

If participants failed to make a response, that trial was considered a missed trial and was excluded from analysis. This surmounted to a small proportion of the data for Experiments 2 (0.6%) and 3 (1.07%). In experiment 1, there were 16.33% of such trials. This higher proportion can be explained by the Single Visual condition’s unaware trials where participants did not respond because they had not detected the visual target). For Experiment 1 in which the dependent variable was reaction time, responses that were faster than 200 ms after target onset were considered anticipatory and were excluded from the analysis; this was the case for 3.3% of all trials of Experiment 1.

### 2.4. Analysis

In Experiment 1, the main measure was reaction time. In Experiments 2 and 3, the task was to localise a visual target and therefore the main analysis involved accuracy data. In all three experiments, the frequency of aware responses was compared with relevant conditions; this was carried out using paired samples *t*-tests.

In the analysis of reported subjective awareness, the PAS responses 2—“brief glimpse”, 3—“almost clear experience”, 4—“clear experience” were grouped into the category of aware responses. They were grouped in this manner because comparing PAS responses between participants can be problematic as individuals may vary in their tendency to use the upper and lower end of the scale due to individual bias (e.g., reporting a very clear experience in an instance where another subject might deem that they only saw a glimpse of the target) [26]. Furthermore, because we used subthreshold visual targets, there were fewer responses at the higher end of the PAS scale (response options 3–4) and this resulted in an unequal number of responses across the four responses available along the entire scale. We therefore decided to collapse all responses that indicated some level of awareness (PAS 2–4), and considered them to all fall under the grouped category of “aware” responses for the analysis.

The Greenhouse-Geyser correction was used where the assumption of sphericity was violated in repeated-measures ANOVAs. Multiple comparisons were corrected with the Bonferroni correction. For analysis sections with multiple *t*-tests (not including post-hoc analysis), the *p*-values were corrected with the Holm-Bonferroni method [27].

### 2.5. Justification of Sample Size

Twenty-three participants took part in Experiment 1, which served as a pilot of the study; we did not conduct a power calculation of this experiment ahead of data collection. Experiment 2 and 3 had 25 participants as a power calculation using G*power, which showed that for an analysis for F-tests with an effect size of 0.25, an B of 0.825 would require a sample size of 25 [28].

## 3. Experiment 1

The approach of experiment 1 was to measure changes in behaviour in relation to unisensory or multisensory events using a classic multisensory RSE paradigm with partial CFS cover of the visual stimulation. The purpose of this was to test whether an auditory signal could help bring a weak visual target into the observers’ awareness with expected behavioural benefits in audio-visual stimulation related to the three principles of multisensory integration [1,5].

The behavioural changes were measured in reaction times and the incidence of reported subjective awareness of visual targets were recorded under three stimulus conditions, namely Single Visual, Visual + Audio, and Single Audio targets each containing 100 trials. In addition, there were 100 catch trials, where no audio or visual stimuli were presented. We expected there to be faster responses in the Visual + Audio condition compared to the Single Audio. We expected the slowest performance in the Single Visual condition, as previous findings have shown that a single weak visual target presented under a CFS mask often goes unnoticed, and that if reaction time response is made to its detection it tends to be slow [29].

### 3.1. Participants and Procedure

Twenty-three naïve participants took part in experiment 1 (18 F, age range 22–40 years, *M* = 26.52, *SD* = 3.68). Thirteen of the participants were right-eye dominant and 22 were right-handed. These demographics describes the sample after one participant was removed for not complying with the task instructions.

The temporal structure of the trials was as follows. The CFS began at the start of the trial and played from start until the period of 1500–3000 ms where the targets could appear. Targets were presented for 100 ms. After the target had been displayed, participants had a window of 1500 ms to respond before the trial timed out (Figure 1a). After each trial, participants were asked to report their awareness of the visual target. The awareness response was not timed. The experiment took approximately 60 min and was split into five blocks of equal length.

### 3.2. Results Experiment 1

#### 3.2.1. Overall Reaction Time by Sensory Stimulation

**Figure 2:**
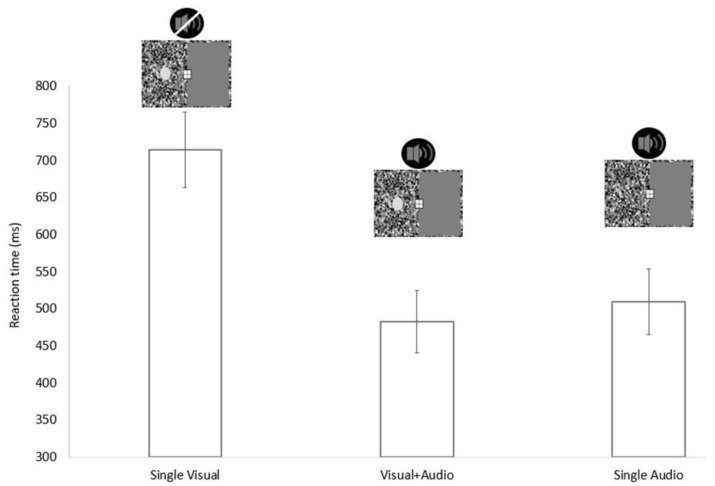
The overall mean of all participants’ individual median reaction times for the three experimental conditions of experiment 1. The error bars represent 95% CI.

Participants’ median reaction times were compared by means of a repeated measures ANOVA between the Single Visual (*M* = 715 ms, *SD* = 125 ms), Visual + Audio (*M* = 483 ms, *SD* = 104 ms), and Single Audio (*M* = 510 ms, *SD* = 109 ms) conditions, without grouping trials by awareness response. This analysis revealed that sensory stimulation had a significant effect of on reaction time (*F* (1.154, 25.38) = 52.335, *p* < 0.001, np2 = 0.704, Greenhouse-Geisser corrected; Figure 2). Paired samples t-tests revealed that the responses in Visual + Audio were significantly faster than both the Single Visual (t (22) = 8.191, *p* < 0.001, Cohen’s d = 2.02) and Single Audio conditions (*t* (22) = −2.79, *p* = 0.011, Cohen’s d = 0.24). Single Audio had significantly faster responses than the Single Visual condition (*t* (22) = 6.656, *p* < 0.001, Cohen’s d = 1.76).

#### 3.2.2. Reaction Time by Awareness

**Figure 3:**
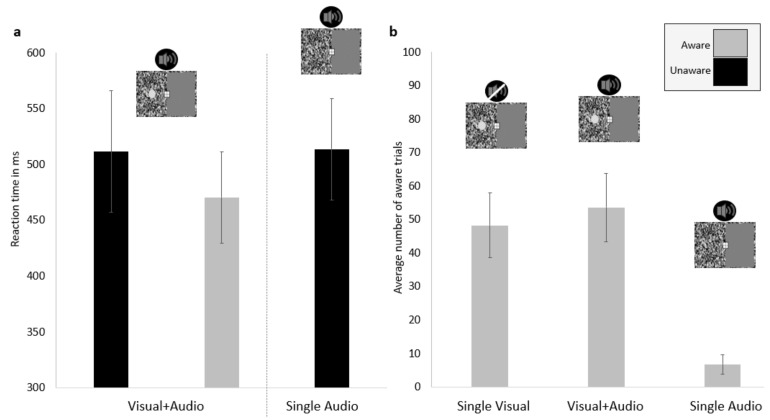
Reaction times of the Visual + Audio and the Single Audio conditions (**a**). The average number of aware trials for the Single Visual, Visual + Audio, and Single Audio conditions (**b**). The error bars represent 95% CI.

In order to investigate the effect of the combined audio and visual stimuli on the incidence of aware responses, the data were split into aware and unaware trials where the Visual + Audio and Single Audio Unaware were compared. The Single Visual condition was excluded from these reaction time comparisons because the overall reaction time for that condition almost exclusively consisted of aware trials, as someone pressing the key to indicate that they had seen a visual target but reporting to be unaware would be an error, which was very infrequent in the sample–1.16% of all valid trials for the Single Visual condition (number of instances: *M* = 0.57, *SD* = 0.84). Furthermore, Aware Single Audio trials were also excluded in the analysis of reaction time awareness as these trials were considered erroneous responses (as the participant indicated to have seen the visual target in a trial when it was not presented). These trials were also relatively rare, with only 6.93% of all valid trials for the Single Audio condition (incidence: *M* = 6.65, *SD* = 7.06). However, both the Single Visual and Single Audio were included in the analysis below regarding the frequency of reporting awareness of the visual target. This analysis showed that participants reported being aware of the visual target more often in the Visual + Audio (*M* = 54, *SD* = 25) condition than in the Single Visual condition (*M* = 48, *SD* = 24; *t* (22) = −2.35, *p* = 0.028, Cohen’s d = 0.24; Figure 3b).

When comparing the reaction times for the sensory stimulation split by awareness, the alpha was adjusted according to the Holm-Bonferroni method for multiple comparisons. Reaction times in Visual + Audio Aware trials (*M* = 470 ms, *SD* = 100 ms) were significantly faster than in the Single Audio Unaware (*M* = 513 ms, *SD* = 111 ms; *t* (22) = −3.913, *p* = 0.001, Cohen’s d = 0.41) and Visual + Audio Unaware (*M* = 511 ms, *SD* = 133 ms; *t* (22) = 2.592, *p* = 0.017, Cohen’s d = 0.35) trials. Reaction time did not differ between Visual + Audio Unaware and Single Audio Unaware trials (*t* (22) = −0.229. *p* = 0.821, Cohen’s d = 0.02; Figure 3a).

### 3.3. Summary of Experiment 1

The results from experiment 1 showed that the combined audio-visual stimuli led to faster responses compared to either modality alone. Reaction time in the Visual + Audio condition was influenced by the subjective awareness, with aware trials being associated with faster RTs. Interestingly, reaction times in the Visual + Audio condition did not differ from the Single Audio condition when participants reported being unaware of the visual stimulus. These findings are also consistent with the association between subjective awareness and reaction time under CFS with previous findings indicating that for unimodal visual RSE to occur, the participant need to report some level of awareness of masked targets [29]. More importantly, the incidence of aware trials was higher for the combined audio and visual stimuli than for the visual stimulus alone, again contributing to the evidence of the possibility that increased reported awareness is caused by multisensory integration. It seems unlikely that the rise in reported awareness was due to an increase in bias for reporting awareness from the presence of the tone, as the Single Audio condition had a remarkably scarce number of trials where participants reported to have seen the visual target. Thus, it seems that the tone instead aided in bringing the visual target into awareness when it was presented.

The underlying mechanism behind these behavioural effects associated with the presence of a tone is however unclear from the findings of experiment 1. However, based on findings from a similar paradigm by Aller et al. [5], we hypothesise that the most likely explanation behind these behavioural effects is multisensory integration. One way to establish if the increased instances of awareness are due to multisensory integration or to some effect of increased arousal is to make use of the principle of spatial congruency in multisensory integration [1]. According to the principle of spatial congruency in multisensory integration, sensory signals need to spatially coincide to be integrated. This effect was examined in Experiment 2 in conditions where we manipulated the spatial relationship between the visual target and the auditory tone in a 4AFC localisation task.

## 4. Experiment 2

In Experiment 1, we found that the simultaneous onset of both visual and auditory stimuli resulted in increased incidence of reported awareness and faster reaction times. In the second experiment, we began to investigate the underlying mechanism behind the behavioural benefits seen in Experiment 1. Increased awareness of masked visual targets under CFS with multisensory stimulation has previously been attributed to multisensory integration [5]. This also fits with the principles of multisensory integration [1] and it is therefore possible that the improved performance in the Visual + Audio condition of Experiment 1 was a result of multisensory integration. Previous findings have also shown that multisensory stimuli coming from a congruent spatial location yielded faster reaction times compared to incongruently located signals [13]. We set out to test if this was the case by manipulating the spatial overlap of visual and auditory signals based on the spatial principle of multisensory integration [1,16] under CFS. Experiments 2 and 3 have a 4AFC design for the visual target location, which allows for a more detailed view of the perceptual experience of participants and their task performance because, arguably, the task is more difficult than Experiment 1 in which there was only one location where the visual target could appear. This more difficult paradigm could, for example, serve as stronger evidence of multisensory interactions for unconscious visual processing if the observer reports not having seen a visual target but is performing above chance in the detection task, which has been seen in hemianopia and neglect patients under multisensory stimulation [9] and also in healthy observers [5].

We hypothesised that if multisensory integration was the cause for the observed behavioural effects, then conditions with spatially congruent visual and auditory signals should have the higher performance in location accuracy and a higher instance of reported awareness of the visual target than conditions in which visual and auditory signals were not spatially congruent. If, however, the cause was of a more general nature (e.g., arousal, alerting) [18,19,20,21], then spatial congruency should not have much of an effect.

### 4.1. Participants and Procedure

Experiment 2 had 25 participants (20 F, age-range 18–38, *M* = 23.68, *SD* = 4.98, 24 right-handed, 15 right-eye dominant) recruited, with 19 receiving course credits for their participation.

An additional ten participants were excluded from the sample. Two did not pass the audio location screening (without the presence of the visual targets or mask, the tones were played from the top left, top right, bottom right, and bottom left and the participant had to locate the tone with keypresses with a minimum accuracy of 75%), six participants reported to be aware of visual targets in at least half of the catch trials (where there was no visual target, only the tone playing), and two participants never reported to be aware of the visual target and could therefore not be included in the analysis.

The experimental task was to indicate in which of the four quadrants the visual target may have appeared. Participants were also told that there could be an auditory tone playing at the same location as the visual target, or elsewhere, but their task was to only locate the visual target. They were also asked to report their subjective awareness using the PAS scale on a trial-by-trial basis. There were four conditions (see Figure 1d): Congruent (the visual and auditory signals were presented from the same quadrant), Incongruent (the tone was played at a mirrored location with respect to the vertical meridian at the centre of the screen), Central (the tone was played from all four speakers, so that it would be localised to the centre of the screen), and No Audio (only the visual target was displayed). The conditions were presented 200 times in total over the course of 10 blocks. For each condition the visual target appeared in each of the four quadrants 50 times. There were in total 880 trials, including 80 catch trials in which the tone was presented but there was no visual target.

At the beginning of each trial, the CFS was presented for a random duration from 1000 to 2500 ms during which a visual target may be displayed for 100 ms duration. After this, the CFS continued for a further 1500 ms until the end of the trial. Participants were instructed not to respond until the trial had stopped, at which point they indicated the spatial position of the probable visual target during a response window of 1000 ms. If no response was made before this time the trial was logged as a missed trial. After reporting the location, they verbally reported their subjective awareness of the visual target (not timed). Each participant completed a short practice block of this task before continuing to do the full experiment. The experiment took approximately 2 h.

### 4.2. Results of Experiment 2

There were four types of responses that could be generated in the experiment. On any given trial, the participant could be correct in locating the visual target and reporting awareness, they could be correct and report to be unaware, they could be incorrect and report to be aware, and they could be incorrect and report to be unaware. The group proportions of these trials are reported in Table 1. For the analysis, the proportion of these types of trials were calculated for each participant.

#### 4.2.1. Correct Detection

**Figure 4:**
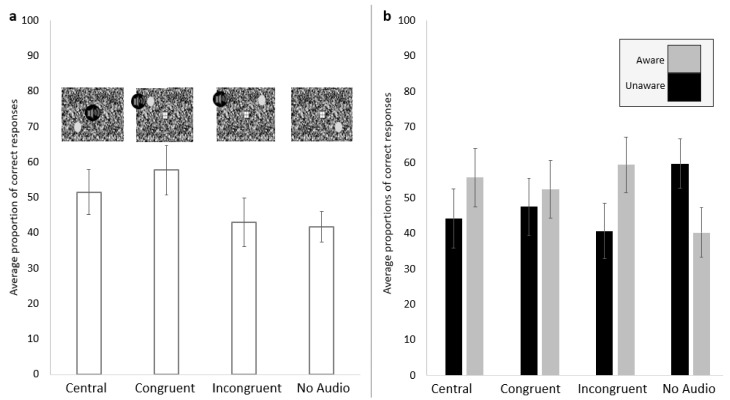
The proportion of correct trials (aware and unaware) for each condition (**a**) and the proportion of aware (grey) and unaware (black) of all the correct trials (**b**).

The proportions of correct detection (aware + unaware) across the four experimental conditions were compared with a repeated measures ANOVA, where the effect of spatial alignment was significant (*F* (2.049, 49.182) = 26.294, *p* < 0.001, np2 = 0.523, Greenhouse-Geisser corrected; Figure 4a). This was followed with paired samples *t*-tests, which revealed that the Congruent condition (*M* = 58.28%, *SD* = 18%) had significantly more correct detections than the Central condition (*M* = 51.83%, *SD* = 16.29%, *t* (24) = −3.796, *p* < 0.001, Cohen’s d = 0.38), the Incongruent condition (*M*= 43.46%, *SD* = 17.78%, *t* (24) = 5.225, *p* < 0.001, Cohen’s d = 0.83), and the No Audio condition (*M* = 42.29%, *SD* = 11.31%, *t* (24) = −6.965, *p* < 0.001, Cohen’s d = 1.06). The Central condition also had more correct detections compared to the Incongruent (*t* (24) = 5.105, *p* < 0.001, Cohen’s d = 0.5) and the No Audio condition (*t* (24) = −6.179, *p* < 0.001, Cohen’s d = 0.68). There was no significant difference between the proportion of correct trials between the Incongruent and the No Audio conditions (*t* (24) = −0.548, *p* = 0.589, Cohen’s d = 0.08).

#### 4.2.2. Awareness of the Visual Target

The proportions of correct + aware trials differed between the four experimental conditions (*F* (1.801, 43.219) = 25.853, *p* < 0.001, np2 = 0.519, Greenhouse-Geisser corrected; Figure 4b). The Congruent (*M* = 32.73%, *SD* = 19.67%) condition had a higher proportion of correct + aware trials compared to the Incongruent (*M* = 27.66%, *SD* = 17.79%; *t* (24) = 3.502, *p* = 0.002, Cohen’s d = 0.27) and the No Audio condition (*M* = 18.12%, *SD* = 10.8%; *t* (24) = −5.815, *p* < 0.001, Cohen’s d = 0.92). The Central condition (*M* = 31.36%, *SD* = 18.43%) had a significantly higher proportion of correct + aware trials compared to the Incongruent (*t* (24) = 3.146, *p* = 0.004, Cohen’s d = 0.2) and No Audio conditions (*t* (24) = −5.922, *p* < 0.001, Cohen’s d = 0.88). There was no significant difference between the Congruent and the Central conditions (*t* (24) = −1.153, *p* = 0.26, Cohen’s d = 0.07). The Incongruent condition had a higher proportion of correct + aware trials compared to the No Audio condition (*t* (24) = −4.798, *p* < 0.001, Cohen’s d = 0.65).

#### 4.2.3. Unconscious Vision

The number of correct + unaware trials per experimental condition might be an indication of unconscious processing of visual information. We determined the chance level performance for each participant by adding the correct + unaware and the incorrect + unaware, and dividing the sum by four (representing the four location options). This individual chance level per condition was then compared to the number of correct + unaware responses for the corresponding condition by means of paired samples *t*-tests. Detection performance was above chance in the No Audio (observed: *M* = 47.68 n, *SD* = 14.68 vs. chance *M* = 38.09, *SD* = 5.61; *t* (24) = 3.788, *p* = 0.001, Cohen’s d = 0.86), the Central (observed: *M* = 40.68 n, *SD* = 15.96 vs. chance *M* = 30.27, *SD* = 9.44; *t* (24) = 4.606, *p* < 0.001 Cohen’s d = 0.79), and the Congruent (observed: *M* = 50.56 n, *SD* = 21.39 vs. chance *M* = 30.2, *SD* = 10.02; *t* (24) = 5.747, *p* < 0.001 Cohen’s d = 1.22) conditions, but not in the Incongruent condition (observed: *M* = 31.28 n, *SD* = 14.59 vs. chance *M* = 31.57, *SD* = 9; *t* (24) = −0.117, *p* = 0.908, Cohen’s d = 0.02).

When comparing the proportion of correct responses for unaware trials between the audio-visual conditions, we found that there was a higher proportion of correct + unaware trials in the Congruent condition (*M* = 25.55%, *SD* = 10.92%) compared to the Central condition (*M* = 20.47%, *SD* = 8.03%; *t* (24) = −4.062, *p* = 0.001, Cohen’s d = 0.53). The Central condition also had a higher proportion of correct + unaware trials compared to the Incongruent (*M* = 15.8%, *SD* = 7.38%) condition (*t* (24) = 3.821, *p* = 0.001, Cohen’s d = 0.61).

It is also possible that when participants were unsure of the location of the visual target, they were more likely to select the location of the tone rather than any of the other two location options. To test if there was a response bias from the tone location, we conducted an analysis of the errors made in the Incongruent condition. We found that when participants made an erroneous location response (i.e., not selecting the location of the visual target) they were above chance in selecting the auditory location over the other two available options both for overall (aware + unaware, *M* = 41.25%, *SD* = 8.82%; *t* (24) = 4.507, *p* < 0.001, against the chance level of three error options 33.3%) and for only unaware trials (*M* = 40.96%, *SD* = 8.57%, *t* (24) = 4.47, *p* < 0.001, against the chance level of three error options 33.3%).

#### 4.2.4. Multisensory Presentation Associated with Increased Awareness

To test if the presence of a tone was associated with a higher proportion of aware trials, all the audio-visual conditions were compared to the No Audio condition. For each participant and condition, the proportions of correct + aware and incorrect + aware were added to obtain the total number of aware trials. The total proportion of aware trials of the audio-visual conditions were compared against the total proportion of aware trials of the No Audio condition. To correct for multiple comparisons, the alpha level was adjusted according to the Holm-Bonferroni method. This analysis revealed that there were in total fewer aware trials for the No Audio (*M* = 22.86%, *SD* = 11.02%) condition compared to the Central (*M* = 39.08%, *SD* = 18.93%; *t* (24) = −6.1, *p* < 0.001 Cohen’s d = 1.05), Congruent (*M* = 39.04%, *SD* = 20.27%; *t* (24) = −5.575, *p* < 0.001 Cohen’s d = 0.99), and Incongruent conditions (*M* = 36.28%, *SD* = 18.11%; *t* (24) = −5.437, *p* < 0.001 Cohen’s d = 0.9).

### 4.3. Summary of Experiment 2

Overall, the findings of experiment 2 show that both detection and awareness are improved by the spatial congruency of the audio-visual stimuli. However, it was also found that for the proportion of correct trials, when the participants reported to be unaware of the visual target there was a significantly higher location accuracy in the Congruent condition compared to the Central (non-informative cue). This might be explained by participants guessing the location of the tone when faced with making a decision without any clear awareness of the location of the visual target. The lack of a difference in correct and aware performance between the Congruent and Central condition could indicate that it is possible that the behavioural benefits observed were not due to multisensory integration per se but may be better explained by a rise in phasic alerting [18,20]. Thus, in reviewing the findings of experiment 2, there is further evidence that the processing of the visual target benefits from the simultaneous onset of an auditory tone. However, our hypothesis was that the Congruent condition would be singled out as having the highest performance, but the findings show a similar accuracy between the Central and the Congruent condition for aware trials (while the Congruent condition outperformed the Central condition for unaware trials). This is somewhat puzzling and does not clearly indicate if the improvement in behavioural performance for the audio-visual conditions is best explained by multisensory integration [1] or an increase in phasic alertness [18,19,20,21]. In order to address this, we conducted a further experiment where we temporally separated the auditory and visual presentations. We hoped that the principle of temporal congruency in multisensory integration could help determine the underlaying cause behind multisensory integration [1].

## 5. Experiment 3

Overall, the findings of experiment 2 showed behavioural benefits from audio-visual stimulation, with the best performance in the Congruent and Central conditions. The Incongruent condition solely outperformed the No Audio condition in detection accuracy when participants reported to be aware of the visual target. However, there was a similar level of benefit in terms of correct detection and reported awareness of the visual target when the sound was spatially congruent and when it was non-informative (central) in relation to the visual target, but when participants reported to be unaware of the visual target performance, it was better for the Congruent condition. Considering these findings in experiment 2, it is still unclear what the likely underlying process behind the behavioural benefits in multisensory stimulation is. If the effect was due to multisensory integration, then we would expect the Congruent condition to have better performance based on the spatial principle of multisensory integration [1], but if the effect was instead due to alertness caused by the tone arousing the system and ‘readying’ it for processing by indicating that there is a visual target about to be displayed, then the performance is expected to be similar across all the audio-visual conditions. To address this, we conducted a third experiment in which the visual target and the tone were temporally separated but the tone was the same as the Central condition of Experiment 2, always coming from the centre of the display. We hypothesised that if performance is better when the signals have a simultaneous onset, then the behavioural benefits are likely to be due to multisensory integration [1]. On the other hand, if the performance of the Simultaneous condition and the condition where the auditory target precedes the visual target are similar, then an increase in alertness is the most probable explanation for the underlying mechanism behind the behavioural benefits of multisensory stimulation [18,19,20,21] under CFS.

### 5.1. Participants and Procedure

Twenty-five participants (17 female) aged 17–55 (*M* = 24.2, *SD* = 7.72), took part. All participants took part on a voluntary basis. Twenty of the participants were right-handed and 20 were right eye dominant. An additional four participants were removed for reporting awareness in 50% or more of the catch trials (i.e., reporting to have seen a visual target when there was none).

The procedure was the same as that of Experiment 2. Participants were instructed to press a key to indicate the location of the visual target in one of the four quadrants as soon as the trial ended. After making the keypress, they were asked to report their subjective experience of the visual target according to the PAS scale. There were four conditions: Audio-Before-Visual: the auditory tone was presented 400 ms before the onset of the visual target; Simultaneous onset of the visual and auditory signals; Visual-Before-Audio: the tone played 400 ms after the onset of the visual target; and No Audio: where only the visual target was displayed (Figure 1e). There were 200 trials for each condition, randomly presented in 10 blocks. As in in Experiment 2, there were also 80 catch trials spread across the 10 blocks and the participants completed a short practice block of the task. The experiment took approximately 2 h.

### 5.2. Results of Experiment 3

As in experiment 2, there were four possible responses: correct + aware, correct + unaware, incorrect + aware, and incorrect + unaware. See Table 2 for the proportions of these types of trials for each condition. For the analysis, the proportion of these types of trials were calculated for each participant.

#### 5.2.1. Correct Detection

**Figure 5:**
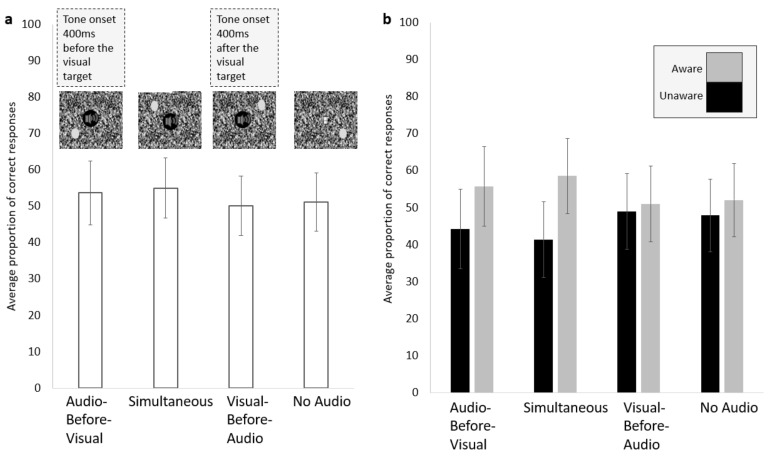
The proportion of correct trials (aware and unaware) for each condition (**a**) and the proportion of aware (grey) and unaware (black) of all the correct trials (**b**). Error bars represent the standard error of the mean.

The proportion of overall correct detection (aware + unaware) was calculated for each participant and then compared between the four conditions: Audio-Before-Visual (*M* = 55.71%, *SD* = 23.3%), Simultaneous (*M* = 55.32%, *SD* = 21.33%), Visual-Before-Audio (*M* = 51.17%, *SD* = 20.81%), No Audio (*M* = 51.99%, *SD* = 20.45%). A repeated measures ANOVA revealed that there was an effect of temporal relationship of the signals on the proportion of correct detection (*F* (2.003, 48.082) = 6.844, *p* = 0.002, np2 = 0.222, Greenhouse-Geisser corrected; Figure 5a). This was followed by paired samples *t*-tests, which revealed that there was no significant difference in correct detection between the Audio-Before-Visual and the Simultaneous conditions (*t* (24) = 0.279, *p* = 0.783, Cohen’s d = 0.02) or between the Visual-Before-Audio and No Audio (*t* (24) = −0.963, *p* =0.345 Cohen’s d = 0.04) conditions. However, correct detections were higher in the Audio-Before-Visual condition than in the Visual-Before-Audio (*t* (24) = 3.522, *p* = 0.002, Cohen’s d = 0.21) and No Audio (*t* (24) = 2.207, *p* = 0.037, Cohen’s d = 0.17) conditions. Similarly, correct detections in the Simultaneous conditions were higher than in the Visual-Before-Audio (*t* (24) = 4.008, *p* = 0.001, Cohen’s d = 0.2) and No Audio (*t* (24) = 3.326, *p* = 0.003 Cohen’s d = 0.16) conditions.

#### 5.2.2. The Visual Target Brought into Awareness

A repeated measures ANOVA revealed that the proportion of correct + aware trials depended upon the temporal relationship between the visual and auditory target (*F* (2.127, 51.042) = 13.522, *p* < 0.001, np2 = 0.36, Greenhouse-Geisser corrected; Figure 5b). This was followed up with paired samples *t*-tests, which showed that the proportion of correct + aware trials in the Audio-Before-Visual (*M* = 36.36%, *SD* = 27.1) and the Simultaneous (*M* = 36.8%, *SD* = 26.08) conditions (*t* (24) = −0.324, *p* = 0.749, Cohen’s d = 0.02), and the Visual-Before-Audio (*M* = 30.46%, *SD* = 24.23) and No Audio conditions (*M* = 31.16%, *SD* = 24.27) were not significantly different from one another (*t* (24) = −1.076, *p* = 0.293, Cohen’s d = 0.03). The proportion of correct + aware trials in the Audio-Before-Visual condition was significantly higher than in the Visual-Before-Audio (*t* (24) = 4.194, *p* < 0.001, Cohen’s d = 0.23) and No Audio (*t* (24) = 3.257, *p* = 0.003, Cohen’s d = 0.2) conditions. They were also higher in the Simultaneous condition than in the Visual-Before-Audio (*t* (24) = 5.183, *p* < 0.001, Cohen’s d = 0.25) and No Audio (*t* (24) = 4.328, *p* < 0.001, Cohen’s d = 0.22) conditions.

#### 5.2.3. Unconscious Vision

Next, to test for evidence of an unconscious interaction between audio and visual processing, the chance level detection performance for each participant and condition was calculated and compared to the actual proportion of their correct + unaware trials. The proportion of correct + unaware trials was above the calculated chance level in all conditions: Audio-Before-Visual (observed: *M* = 37.56 n, *SD* = 15.35; chance: *M* = 27.9, *SD* = 12.3; *t* (24) = 4.52, *p* < 0.001, Cohen’s d = 0.69), Simultaneous (observed: *M* = 36.72 n, *SD* = 16.37; chance: *M* = 27.71, *SD* = 12.12; *t* (24) = 4.59, *p* < 0.001 Cohen’s d = 0.63), Visual-Before-Audio (observed: *M* = 40.56 n, *SD* = 16.97; chance: *M* = 31.44, *SD* = 11.22; *t* (24) = 3.644, *p* = 0.001, Cohen’s d = 0.63), and No Audio (observed: *M* = 40.96 n, *SD* = 17.32; chance: *M* = 30.66, *SD* = 11.29; *t* (24) = 4.113, *p* < 0.001, Cohen’s d = 0.71). The two conditions that would be best suited to answer the question of whether multisensory integration or increased phasic alertness best explained the data were the Audio-Before-Visual and the Simultaneous conditions. If the accuracy between these two was equal, then it is likely that an increase in phasic alertness best explained the data, whereas if the Simultaneous condition outperformed the Audio-Before-Visual, then the most likely underlaying mechanism is multisensory integration. To this end, the proportion of correct + unaware trials for the two were compared. There was no significant difference in the proportion of unconscious + correct trials between the Audio-Before-Visual (*M* = 19.35%, *SD* = 7.57%) and Simultaneous (*M* = 18.52%, *SD* = 8.33%; *t* (24) = 1.044, *p* = 0.307, Cohen’s d = 0.1).

#### 5.2.4. Multisensory Presentation Associated with Increased Awareness

Finally, the proportion of total aware trials were compared across conditions. These entailed the total proportion of trials where a participant reported to be aware regardless of whether they were correct or not in locating the visual target, as these were added together and compared across conditions. First, these were all compared to the No Audio condition to test whether findings from experiment 1 with a higher tendency to report awareness of the visual target increased with the presence of the tone. In experiment 3, there was no consistent support for an increased tendency in reporting awareness of the visual target with the presence of a tone because the No Audio (*M* = 37.47%, *SD* = 23.23%) condition had a significantly smaller proportion of aware trials compared to the Audio-Before-Visual (*M* = 42.57%, *SD* = 24.54; *t* (24) = 3.657, *p* = 0.001, Cohen’s d = 0.21) and Simultaneous (*M* = 44.01%, *SD* = 24.8%; *t* (24) = 4.394, *p* < 0.001, Cohen’s d = 0.27) conditions. Although there were on average fewer aware responses to the Visual-Before-Audio condition (*M* = 35.65%, *SD* = 22.58%) compared to the No Audio, this difference was not significant (*t* (24) = −2.004, *p* = 0.057, Cohen’s d = 0.08), which indicates that the presence of the tone alone is not sufficient to bias participants to report awareness of the visual target, but certain temporal relationships do need to be met for an increase in awareness of the visual target to arise. The proportion of aware trials in Audio-Before-Visual and Simultaneous conditions were not significantly different from one another (*t* (24) = −1.25, *p* = 0.223, Cohen’s d = 0.06). The statistical tests of this section had their alpha levels adjusted according to the Holm-Bonferroni method to correct for multiple comparisons.

### 5.3. Summary of Experiment 3

The most crucial comparison to be made in experiment 3 was between the Audio-Before-Visual and Simultaneous conditions, as these are the conditions that can best help distinguish between whether the underlying mechanism behind behavioural benefits seen in multisensory stimulation is best explained by multisensory integration or an increase in alertness. There was no difference in correct detection overall or separated by awareness between the Audio-Before-Visual and Simultaneous condition, however both outperformed the Visual-Before-Audio and No Audio condition. The Visual-Before-Audio and No Audio conditions were not significantly different in terms of correct detection or frequency of aware responses, which indicates that the tone increased awareness of the visual target only when it preceded or was simultaneous with the onset of the visual target.

## 6. Discussion

The main finding across the three experiments was that the presence of a tone aided in bringing subthreshold visual targets into awareness, irrespective of whether the tone was task-relevant. This was, however, only the case when the onset of the tone was simultaneous to or preceded the visual target. Thus, the findings of the current study replicated the general body of evidence in that information from one sensory modality can aid in the processing of a target in another modality [1,2,3,4,5]. In Experiment 1, we replicated the effect of the multisensory RSE paradigm in that multisensory stimulation yielded faster responses and higher instances of reported awareness of the visual target. We then conducted Experiments 2 and 3 to address the question of whether the mechanism behind the behavioural benefits in multisensory stimulation are most likely due to multisensory integration or a rise in phasic alertness. In Experiment 2, we hypothesised that the detection performance for reporting the location of a visual target presented at the detection threshold would be superior for the Congruent auditory-visual condition, both in terms of detection accuracy and for the proportion of aware trials compared to Incongruent, non-informative audio (Central), and No Audio conditions. If this was the case, then the most probable explanation would be that multisensory integration was mediating the effect. We found that overall, the performance in the Congruent condition was superior to the other spatial alignments of targets in location accuracy. However, the Congruent and Central (non-informative auditory stimulus) conditions did not significantly differ in the proportion of correct trials in which the participant reported to be aware of the visual target. The Congruent condition, however, had a significantly higher proportion of correct detection for unaware trials compared to the Central condition.

The Congruent and Central conditions both outperformed the Incongruent condition in terms of accuracy. All audio-visual conditions of Experiment 2 had a higher proportion of trials where participants were correct and reported to be aware of the visual target compared to the No Audio condition. Although the presence of the tone was associated with more correct and aware trials, there was a discrepancy in performance between the Incongruent and the other two audio-visual conditions in that the Incongruent condition had significantly worse performance compared to the other two. This finding is in agreement with previous experiments with spatial separation of audio-visual targets and the spatial principle of multisensory integration [1,3] as the spatial relationship between the targets appeared to be instrumental in the proportion of correct and correct + aware trials for each condition. Furthermore, the findings suggest that multisensory integration is a contributing underlying mechanism behind the changes in performance under multisensory stimulation. However, considering the similarities in the proportion of correct + aware trials found in the Congruent and Central condition, it is likely that phasic alertness is an important factor in mediating behavioural change in multisensory stimulation [20,21]. The findings of Experiment 3 are further in support of alertness being the main underlying mechanism. The hypothesis for Experiment 3 was that if the performance was equal in the conditions where the auditory signal onset preceded the visual signal by 400 ms (Audio-Before-Visual) or was simultaneous with the visual target onset, then the effect was most likely due to alertness rather than multisensory integration alone, which would also explain the similarities found between the Congruent and Central conditions in experiment 2. In Experiment 3, we found that there was very similar performance between the Audio-Before-Visual and Simultaneous condition for both the overall proportion of correct detection, and correct detection with aware trials and in unconscious vision. The temporal relationship between auditory and visual targets is important, as demonstrated in Experiment 3. When the auditory signal occurred 400 ms after the visual target (Visual-Before-Audio condition), the performance was no different from the No Audio condition. The presence of the tone was only associated with increased awareness of the visual target when the tone was presented simultaneously with or before the visual target. Thus, considering the findings of Experiments 2 and 3, the best explanation seems to lie in a rise in phasic alertness as the main contributor in a subthreshold visual target breaking through suppression and reaching awareness in healthy observers. These findings are also in alignment with previous reports regarding precision in temporal judgement in multisensory stimulation most likely stemming from alertness rather than temporal expectations [19].

The Congruent condition did outperform the Central condition in experiment 2 in overall and unconscious correct detection. This may suggest that multisensory integration is also involved in the process, perhaps more pertaining to unconscious vision. However, a possible explanation for this is that this may simply reflect a response bias to report the location of the tone when no other information is present to help a participant make their decision. This explanation was supported by the error analysis of the Incongruent condition of Experiment 2, where it was found that participants were more likely to select the location of the tone when making an error to a location that was not where the visual target had been presented. This was true for both all Incongruent error trials (aware + unaware) and for Incongruent unaware error trials alone. It is therefore possible that the tone location served as a strong influence when participants selected an erroneous location instead of the visual target location.

The body of literature has overwhelmingly showed that there are behavioural benefits associated with audio-visual stimulation in both healthy observers [1,3,5,6,7,8,19,20,21] and patient groups [2,29,30,31]. However, some aspects of how visual awareness is affected by the temporal relationship between signals has yielded discrepant results, specifically when an auditory signal precedes a visual target. Therefore, in the light of the similar findings of the Audio-Before-Visual and Simultaneous condition in experiment 3, we wish to discuss some disagreeing findings from the attentional blink paradigm. It has been found that when an auditory signal precedes the visual target in the attentional blink paradigms, this can both aid [7] or have no beneficial effect on the conscious detection of a visual stimulus [8]. The rate at which T2 (the second target in the display) escapes the attentional blink with the help of the introduction of an auditory signal appears to depend on the nature of the auditory signal [7,8]. For example, Olivers and Van der Burg [8] used a tone and Adam and Noppeney [7] used spoken letter names in a task where participants were asked to identify T1 (the first target) and T2 (the second target) (which were letters, and in Adam and Noppeney’s experimental task the spoken letters could be congruent or incongruent with the letters of T1 and T2). Both Adam and Noppeney [7], and Olivers and van der Burg [8] found improved detection of T2 when the auditory stimulation (tone and spoken letters) was presented simultaneously with T2. However, their results varied when the auditory stimulation preceded T2. Adam and Noppeney found that presenting a congruent spoken letter at the same time or prior to T2 onset improved T2′s chances of reaching visual awareness and escaping attentional blink. Contrary to this, Olivers and Van der Burg found that T2 identification improved when the tone was presented synchronously with the beep and performance was not improved when the beep preceded the visual target. Adam and Noppeney argued that the effect in their study could be due to an auditory priming that improved the identification performance, while Olivers and Van der Burg suggested that their findings were not due to an alerting effect since there was no improvement in T2 identification when the tone preceded its onset. Although we used a tone that was not informative as to the identity of the visual target, we found no significant difference in the Audio-Before-Visual (which preceded the visual target) and the Synchronous condition of Experiment 3, but they both outperformed the condition where the tone onset was after the visual target (Visual-Before-Audio), which is indicative of the improved performance being caused by increased phasic alerting [19,20]. Hence, our findings are more in agreement with Adam and Noppeney as the preceding auditory signal aided in detection accuracy and was associated with higher subjective awareness, even though we used a tone like Olivers and Van der Burg.

The behavioural benefits of multisensory stimulation in unconscious vision were not supported in Experiment 1, in that the reaction time between the Single Audio and Visual+Audio stimuli were not significantly different when the participant reported to be unaware of the visual target. It is noteworthy that the subjective experience of stimulation was the same in these contexts, as the participants reported to not having seen the visual target. Thus, it seems that the experience of the participant was associated with changes in reaction time rather than what was in fact displayed to them. Moors et al. [32] argued that based on recent findings from CFS studies, it is likely that the perceptual experience of visual stimuli that is suppressed by CFS is sparse and fractioned, pertaining mainly to basic visual features rather than semantic content, with the deep suppression that comes with CFS. Considering this in light of the findings of Experiment 1, when participants report to have not seen the visual target under CFS in the Visual + Audio condition, it is possible that the visual target is not spared enough to be unconsciously processed in any capacity, and this is why we do not observe any evidence of behavioural benefits from the unconscious processing of the visual target (i.e., no significant difference in reaction time between the unaware trials of the Visual + Audio and Single Audio conditions). We urge that future research aim to further assess the relative weights of multisensory integration and alertness in contributing to behavioural benefits from multisensory stimulation, as it is likely that both of these contribute to the process. One possible way to explore this could be to replicate the experimental setup and procedure of the current manuscript with patients who suffer from Hemispatial neglect due to neurological damage to their right hemisphere [18] and compare these data to that of healthy control subjects. This could be of interest considering that left side Hemispatial neglect is associated with disruption to attentional processes [18]. Another possibility would be to measure reaction time data in a setup like Experiment 2 of this manuscript to explore whether spatial congruency could lead to faster responses compared to the non-informative sound (from the centre of the display), even though the performance in terms of accuracy was similar in these two conditions. Computational modelling techniques can then be applied to elucidate the cognitive stages of multisensory processing.

Furthermore, the findings of these experiments could be of relevance in rehabilitation of vision in hemianopic patients [33,34,35,36]. Systematic visual stimulation of areas of blindness using detection and discrimination tasks can lead to increased visual sensitivity within the blind field [37]. There is also evidence that combining auditory and visual stimuli [38] as well as visual and transcranial direct current stimulation [39,40,41] can enhance rehabilitation outcomes. Nevertheless, the mechanisms for such improvements have not yet been established. In case of audio-visual stimuli, based on current findings, one can speculate that transient attention and alerting mechanisms play a significant role in improved detection. It is also possible that combining audio-visual and electrical stimulation may provide more efficacy in achieving positive outcomes. These issues are currently subjects for active investigations.

## 7. Conclusions

In conclusion, we observed expected behavioural effects of multisensory stimulation when a tone was added to the presentation of a subthreshold visual target. That is, the tone aided the visual target in breaking through the suppression of a dynamic Mondrian mask. Furthermore, after separating the auditory and visual signals spatially and temporally to observe changes in performance, we could conclude that alertness is likely the major contributor to the behavioural benefits from multisensory stimulation in subthreshold visual targets reaching awareness under CFS. However, the evidence from spatial separation of the signals is also indicative of multisensory integration being a contributor to the observed behavioural changes.

## Figures and Tables

**Table 1:** Percentage of the types of responses (i.e., correct/incorrect and aware/unaware) per condition for Experiment 2.

Response	Congruent	Central	Incongruent	No Audio
Correct Aware	32.73%	31.35%	27.64%	18.08%
Correct Unaware	25.51%	20.48%	15.79%	24.15%
Incorrect Aware	6.32%	7.71%	8.62%	4.74%
Incorrect Unaware	35.44%	40.47%	47.95%	53.03%

**Table 2:** Percentage of the types of responses (i.e., correct/incorrect and aware/unaware) per condition for Experiment 3.

Response	Audio-Before-Visual	Simultaneous	Visual-Before-Audio	No Audio
Correct Aware	36.11%	36.85%	30.54%	31.27%
Correct Unaware	19.41%	18.43%	20.75%	20.83%
Incorrect Aware	6.22%	7.51%	5.12%	6.37%
Incorrect Unaware	38.26%	37.21%	43.59%	41.54%

## Data Availability

Data available in a publicly accessible repository. The data presented in this study are openly available in Open Science Framework. Access date: 02/06/2022. Doi for the page where the data is stored on the Open Science Framework server: https://osf.io/63w8g/.
